# Genome Sequence of a Bangladeshi Strain of Raoultella ornithinolytica, a Pathogen with Metal and Antimicrobial Resistance Genes Isolated from a Pet Cat

**DOI:** 10.1128/mra.00863-22

**Published:** 2023-04-10

**Authors:** Jahangir Alam, Mridha M. Kamal Hossain, M. Shahdat Hossain, Shamima Ahmed, M. Amimul Ehsan, Rubaya Rubaya, Anjuman Ara Bhuyan

**Affiliations:** a National Institute of Biotechnology, Dhaka, Bangladesh; b Bangladesh Agricultural University, Mymensingh, Bangladesh; University of Maryland School of Medicine

## Abstract

Raoultella ornithinolytica is an emerging pathogen that causes human infections. We report the isolation and genome sequencing of *R. ornithinolytica* from an oral swab of a Persian pet cat in Dhaka, Bangladesh. The genome length was 5,375,160 bp, with 55.9% G+C content. It carries putative genes associated with resistance to antibiotics and metals.

## ANNOUNCEMENT

Raoultella ornithinolytica, belonging to the family *Enterobacteriaceae*, is a Gram-negative, nonmotile, rod-shaped bacterium (1) found in wet soil, fish, insects, and hospital environments (2–4). It has also been isolated from poultry and seafood products (5, 6). Human infections with *R. ornithinolytica* are increasing, and it is considered an emerging pathogen (7, 8). Its potential for becoming multidrug resistant has been reported. Hence, characterization of *R. ornithinolytica* is essential. Here, we present the genome sequence of *R. ornithinolytica* strain nibro01, isolated from an oral swab of a Persian pet cat.

The strain was isolated using a procedure used for testing of Salmonella spp. (9). After 18 h of incubation on MacConkey agar at 37°C, the bacteria produced a creamy white, round colony. PCR with Salmonella plasmid virulence (*spvC*) gene-specific primers (10) generated a ~252 bp product. After sequencing of the PCR product, the data were subjected to a search using the nucleotide BLAST tool (https://blast.ncbi.nlm.nih.gov/Blast.cgi) and found to have 99.60% identity with sequences of *R. ornithinolytica*. The strain was further confirmed as *R. ornithinolytica* using matrix-assisted laser desorption ionization–time of flight (MALDI-TOF) mass spectrometry (MALDI Biotyper; Bruker, Germany) as described earlier (11). Antimicrobial profiling was performed using the disk diffusion method (12).

Genomic DNA was extracted from 10 mL of a culture of strain nibro01 using the PureLink genomic DNA minikit (Invitrogen, USA) according to the manufacturer’s instructions. Genome sequencing was conducted using the Illumina MiSeq platform for library preparation in paired-end, 2 × 150-bp format (Invent Technologies, Bangladesh). FastQC 0.11.9 (13) and MultiQC 1.11 (14) were used to check the quality. Sequence read errors were corrected and the sequence was assembled using Shovill 1.0.4 with SPAdes 2.3 (15). Common adapters and primers were first removed using Trimmomatic with a minimum contig length of 200. Sequences were further annotated with Rapid Annotations using Subsystems Technology (RAST), using the RASTtk annotation scheme (16, 17). Default parameters were used for all software. The total number of sequence reads was 759,428, and the average sequence length was 150. The assembly was ~5.4 Mb long in total and consisted of 145 contigs. The genome had a coverage of 34× and a G+C content of 55.9%, with 5,211 coding sequences and 389 RAST subsystems. The genome had 94 RNAs, including 15 rRNAs, 79 tRNAs, and 1 transfer-messenger RNA (tmRNA). The genome also contained 9 noncoding RNAs (ncRNAs). The *N*_50_ contig size was 89,898 kb, and the *L*_50_ value was 20. The strain contains 52 pseudogenes. The major putative genes are responsible for carbohydrate metabolism (489), amino acid assimilation (456), protein metabolism (249), cofactors, vitamins, prosthetic groups, pigment biosynthesis (201), respiration (124), virulence, disease and defense (62), etc. We further identified putative fosfomycin and fluoroquinolone resistance genes; multidrug resistance efflux pumps; and cobalt, zinc, cadmium, and copper resistance genes. The isolate was found to be resistant to fosfomycin (200 μg), ciprofloxacin (5 μg), imipenem (10 μg), doripenem (10 μg), and ticarcillin (75 μg) but sensitive to gentamicin (10 μg), tobramycin (10 μg), amikacin (30 μg), netilmicin (30 μg), meropenem (10 μg), ceftazidime (30 μg), cefepime (30 μg), cephalexin (30 μg), levofloxacin (5 μg), piperacillin (100 μg), amoxicillin-clavulanic acid (75/10 μg), aztreonam (30 μg), colistin, and polymyxin B.

### Data availability.

The genome sequence of *R. ornithinolytica* has been deposited at GenBank under accession number JAPUBZ000000000. The version described in this paper is the first version. In addition, the raw data were submitted to GenBank under SRA accession number SRP370918.
